# A Study of the Genomic Variations Associated with Autistic Spectrum Disorders in a Russian Cohort of Patients Using Whole-Exome Sequencing

**DOI:** 10.3390/genes13050920

**Published:** 2022-05-20

**Authors:** Ekaterina A. Gibitova, Pavel V. Dobrynin, Ekaterina A. Pomerantseva, Elizaveta V. Musatova, Anna Kostareva, Igor Evsyukov, Sergey Y. Rychkov, Olga V. Zhukova, Oxana Y. Naumova, Elena L. Grigorenko

**Affiliations:** 1 Computer Technologies Laboratory, University of Information Technologies, Mechanics and Optics, Saint Petersburg 197101, Russia; e.gibitova@yandex.ru (E.A.G.); pdobrynin@gmail.com (P.V.D.); evsyukov007@mail.ru (I.E.); 2 Department of Molecular and Human Genetics, Baylor College of Medicine, Houston, TX 77030, USA; 3 Human Genetics Laboratory, Vavilov Institute of General Genetics RAS, Moscow 119991, Russia; sergey.rychkov@gmail.com (S.Y.R.); zhukova@vigg.ru (O.V.Z.); 4 The ‘Genetico’ Center for Genetics and Reproductive Medicine, Moscow 119333, Russia; e.pomerantseva@gmail.com (E.A.P.); musatova@genetico.ru (E.V.M.); 5 Almazov National Medical Research Centre, Saint Petersburg 197341, Russia; akostareva@hotmail.com; 6 Department of Women’s and Children’s Health, Karolinska Institute, Stockholm 17177, Sweden; 7 Department of Psychology, University of Houston, Houston, TX 77204, USA; 8 Department of Psychology, Saint-Petersburg State University, Saint Petersburg 199034, Russia; 9 Center of Cognitive Research, Sirius University of Science and Technology, Sochi 354340, Russia

**Keywords:** Autistic Spectrum Disorder, whole-exome sequencing, Russian cohort, copy number variation

## Abstract

This study provides new data on the whole-exome sequencing of a cohort of children with autistic spectrum disorders (ASD) from an underexplored Russian population. Using both a cross-sectional approach involving a control cohort of the same ancestry and an annotation-based approach involving relevant public databases, we explored exonic single nucleotide variants and copy-number variation potentially involved in the manifestation of ASD. The study results reveal new potential ASD candidate-variants found in the studied Russian cohort and show a high prevalence of common ASD-associated genomic variants, especially those in the genes known to be associated with the manifestation of intellectual disabilities. Our screening of an ASD cohort from a previously understudied population allowed us to flag at least a few novel genes (*IGLJ2*, *FAM21A*, *OR11H12*, *HIP1*, *PRAMEF10*, and *ZNF717*) regarding their potential involvement in ASD.

## 1. Introduction

Autism spectrum disorders (ASDs) represent a broad spectrum of neurodevelopmental conditions characterized by severe impairment in social interactions and communication skills and the manifestation of restricted, stereotypical behaviors. In 50–70 percent of cases, an ASD co-occurs with intellectual disability [1,2,3]. In addition, multiple other comorbid medical, psychiatric, neurological, and psychological conditions are commonly observed in ASD [3,4,5]. Emerging in early childhood, ASD are lifespan disorders that can be highly disabling [2]. ASD are high-incidence disorders. With slight regional variations, a worldwide average of ASD prevalence was estimated at ~7.6 per 1000 or one in 132 persons in 2010 [6]. As new practices for identifying ASD continue to be developed, these estimates tend to increase. For example, in the USA, the 2016 prevalence estimate of 18.5 per 1000 children was 2.8 times higher than that reported in 2000–2002 [7].

The etiology of ASD is not fully understood. Similar to many other developmental disorders, genetic risk, environmental exposure, and their interplay appears to contribute to the causal landscape of ASD [2,8,9,10]. The genetic contribution to ASD is strongly supported by twin and family studies [9,11,12,13]. A recent large-scale multinational population-based investigation has demonstrated a remarkably high heritability of ASD of 80% [10]. The empirical literature provides a growing body of evidence that the genetic etiology of ASD might be linked to a complex architecture of rare, often pathogenic, de novo mutations of large effects and/or the cumulative impact of multiple common variants with small effects [14,15,16]. Both the rare occurrence of the former and the small effect sizes of the latter underlie the main challenges in detecting ASD-associated genetic factors; addressing these challenges would require a considerable increase in sample size, an extension of the research geography, and the involvement of multinational patient cohorts. 

Over the past decade, research on ASD—ASD prevalence, the spectrum variability, and the contribution of environment and genetics to ASD etiology—has actively extended to previously less outreached minority groups: various racial, ethnical, and cultural communities. This research has revealed reduced differences in ASD prevalence for different ethnic cohorts destroying the stereotype of the significant prevalence of ASD in white populations and attributing this difference in prevalence mainly to limited access to healthcare services and screenings for minority communities; see, e.g., [17,18]. While the ASD prevalence can be relatively similar across various ethnic and cultural groups, the frequencies of different syndromes and disorders falling under the ASD umbrella, the disorders’ severity, and comorbid health conditions seem to vary across populations. While the ASD prevalence can be relatively similar across various ethnic and cultural groups, the frequencies of different syndromes and disorders falling under the ASD umbrella, the disorders’ severity, and the patterns of comorbid health conditions seem to vary across populations [19,20,21]. Collectively, the studies reporting on such phenotypic variability in ASD among various ethnic groups have attributed this to differences in both the cultural environment and the genetic background. The latter may shape the population-specific pattern of ASD via (1) elevated frequencies of some common ASD-related genetic variants, e.g., as has been shown for several SNPs in the *SCN2A*, *FOXP1*, and *SYNGAP1* genes in indigenous American populations in the Amazon [22], rare population-specific causal variants, and (3) additive effects of common disease-associated variants and rare, likely pathogenic variants in the genetic background [23].

Thus, exploring ASD-associated genetic variants in various ethnic and geographic populations may increase diversity in reference genetic databases connecting specific variants to ASD and comorbid conditions that, in turn, may significantly improve genetic testing and its interpretation. To fill a ‘geographical’ gap, here we report data from a genomic study of a Russian cohort of children with ASD using exome sequencing.

## 2. Materials and Methods

### 2.1. Participants

The study participants were 193 children with ASD (mean age = 7 ± 4 years; 18 girls and 175 boys), ascertained through the ‘Genetico’ Center for Genetics and Reproductive Medicine (Moscow, Russia). The children were enrolled in a project focused on the genetic screening of ASD in a west-Russian population, based on ASD diagnoses established by child psychiatrists. The diagnostics of ASD in Russia are carried out in accordance with the Clinical Recommendations for Autism Spectrum Disorders (ID:594) issued by the Association of Psychiatrists and Psychologists for Evidence-Based Practice and approved by the Ministry of Health of the RF (https://cr.minzdrav.gov.ru/recomend/594_1, accessed on 15 May 2022). The diagnosis of ASD was established following the Autism Diagnostic Observation Schedule Second Edition (ADOS-II) based on the clinical picture and developmental history characteristics of ASD—a combination of symptoms of qualitative disorders of social interaction, communication, and limited, stereotyped, repetitive behavior. According to the Clinical Guidelines, the following tools to screen for the ASD symptoms were used: Checklist for Autism Spectrum Disorders (CASD), Social Communication Questionnaire (SCQ), and Autism Diagnostic Interview-Revised (ADI-R).

The children’s demographics and comorbid diagnoses, along with the participants’ family histories, are shown in Supplementary Table S1. The data on the clinical features in ASD individuals are summarized in Table 1. These data were collected from the children’s medical records and interviews with parents. In addition, 51 individuals without ASD from the west-Russian general population enrolled via the Almazov National Medical Research Centre (St. Petersburg, Russia) were involved in the study as a comparison group; hereafter, they are referred to as nonASD. The nonASD comparison group included children and adults (mean age = 23 ± 16 years; 29 females and 23 males) who did not have a lifetime or current medical history of ASD, and any neurodevelopmental and psychiatric disorders, as per the interview with the participants and their medical records.

### 2.2. Exome Library Preparation and WES Data Processing

For the sequencing library preparation, three different exome capture assays were utilized. For the ASD cohort, the TruSeq DNA Exome and the SureSelect Human All Exon V7 were used for 73 and 120 individual samples, respectively. Whole-exome sequencing (WES) was performed using the Illumina HiSeq 4000 platform. The library preparation and sequencing were conducted at the ‘Genetico’ Center. WES data for the controls—51 nonASD individuals—were obtained using the SureSelect V6 r2 (hg19 build) capture assay for library preparation followed by sequencing on the Illumina platform. The sequencing was performed at the Almazov National Medical Research Centre. 

Sequencing data quality control was performed using the FastQC v. 0.11.9 [24]. Sequencing reads were aligned using the BWA v. 0.7.17-r1188 [25] to a reference genome depending on the exome enrichment panel utilized: the hg19 (UCSC) was used for the samples prepared with the TruSeq DNA Exome and the SureSelect V6 panels, and the hg38 (UCSC) for the samples prepared with the SureSelect V7 exome capture assay. Subsequently, the aligned sequences were subjected to sorting, deduplication, and base quality score recalibration as per the GATK Best Practices [26]. 

### 2.3. Single Nucleotide Variant Calling

Detection of single nucleotide polymorphisms (SNPs) was performed as follows. First, base recalibrated BAM files were submitted to the Germline short variant (SNPs and indels) discovery workflow implemented in the GATK v. 4.1.6. Second, each sample was submitted to the HaplotypeCaller separately with an appropriate BED file of intervals. Since three different capture assays were used for the sequencing library preparation, the corresponding three datasets were subjected to joint-genotyping separately. Third, the consolidated GVCF files were submitted to an exact test of the heterozygosity excess, applying the hard filtering parameter of ExcessHet > 54.69. The subsequent filtering procedures were performed using the VariantRecalibrator algorithm implemented in the GATK with the following truth sensitivity level parameters: 99.95 and 99.90 for the SureSelect and Truseq ASD-data subsets, respectively, and 99.50 for the nonASD subset. The established thresholds were chosen empirically to increase the possibility of detecting rare variants. Specifically, this allowed the detection of the maximum number of true-positive calls with minimal risk for false-positive calls, as can be seen in the tranches plots presented in Supplementary Figures S1 and S2).

The hg38 SNP call-sets were converted to the hg19 build using the Assembly Converter. Three SNP call-sets were merged using the bcftools v. 1.10.2 [27]; the ‘include-non-variant-sites flag’ function implemented in the GenotypeGVCF pipeline was applied to account for the missing and reference genotypes. The indels were excluded, and the final merged SNP call-set was used in the downstream analysis. The SNP call-set was annotated to several databases: refGene, cytoBand, ClinVar, ExAC, avSNP147, dbNFSP v. 3.0a, and gnomAD Exome, using the ANNOVAR tools [28].

### 2.4. CNV Detection in WES Data

The detection of copy number variations (CNVs) in the WES data was performed by the ‘exomecn.mops’ function implemented in the cn.mops R package v. 1.32.0 [29]. The following parameters were adjusted: priorImpact = 100, upperThreshold = 0.59, lowerThreshold = −0.99, and useMedian = TRUE. Both segmentation algorithms, fastseg [29] and DNAcopy [30], were applied. Sixteen ASD samples were excluded: one sample prepared with the SureSelect V7 panel, due to a low genome coverage; and 15 samples prepared with the TruSeqExome panel, due to a batch-effect related to the use of IDT adapters. The CNV calling was performed in three separate runs for the subsets of samples processed with different exome capture assays for the library preparation—the TruSeq Exome (N = 58 ASD), the SureSelect V7 (N = 119 ASD), and the SureSelect V6 (N = 51 nonASD).

### 2.5. Analysis of ASD-Associated Variants

The association analysis was performed using the PLINK v1.9 toolset [31]. Prior to the association analysis, the SNP call-set underwent linkage disequilibrium (LD) pruning and clumping procedures. The LD pruning was conducted according to the following parameters of the ‘indep’ command: window size = 50, window shift = 5, and VIF threshold = 2. The SNP call-set was adjusted based on the following QC parameters: –maf 0.01, –geno 0.05, –hwe 0.001. After relatedness testing, nine individuals from closely related pairs (PI HAT ≥ 0.125) in the ASD cohort were removed. A case-control association analysis was performed using Fisher’s exact test to generate significance values adjusted for multiple testing using Bonferroni correction. Established candidate SNP variants had to meet inclusion criteria based on the predicted pathogenicity score thresholds: a SIFT score < 0.05 [32] and PolyPhen2 HDIV score ≥ 0.453 [33]. The polymorphisms with unknown pathogenicity were also considered as potential candidate variants. In addition, the SNP and CNV call-sets were intersected with an autism gene database, AutDB [34]. For the CNV call-sets, the intersection with the list of CNVs reported in the AutDB (validated) was performed using the following command: ‘bedtools intersect -a test.bed -b autdb.bed -f 0.70 -r -wa -wb > output.’ For a successful query, a test region had to overlap at least 70% of an AutDB record.

## 3. Results

### 3.1. Genome-Wide SNP Association Analysis

A summary of the variant calling statistics across the comparison groups and the WES-data subsets is shown in Supplementary Table S2. Altogether we detected 237,019 SNPs across all individuals from both comparison cohorts, ASD and nonASD. The mean number of the detected SNPs per individual varied from 23 to 30K depending on the exome enrichment assay applied, which corresponds to the value of ~25K SNPs per individual expected for the exome data [35]. The transition/transversion (Ti/Tv) ratio varied from 2.73 to 2.93, which corresponds to the value of 3.0 observed in the exonic regions [36].

After variant filtering and LD pruning, 22,249 remaining SNPs were included in the case-control association analysis. The association analysis identified ten variants related to eight genes that surpassed the genome-wide significance threshold (Table 2). These ten SNPs were novel variants not previously reported in association with ASD. Moreover, the eight genes harboring these variants also were not found among the over a thousand human genes implicated in ASD as per records in relevant databases—the SFARI [37] and AutDB [34]. According to the SNP functional annotation, the following variants might be highlighted: two likely pathogenic synonymous substitutions in the *IGLJ2* (rs8033) and *HIP1* (rs1167801) genes and five missense variants with moderate or high deleterious effects located in the *PRAMEF10*, *ZNF717*, *FAM21A*, and *OR11H12* genes. The annotation of these genes against the databases on human diseases MalaCards [38] and OMIM [39], and human phenotype ontologies HPO [40], did not reveal associations with ASD and ASD-related phenotypes (Table 2).

### 3.2. CNV Burden in the ASD Cohort Compared to nonASD

**Figure 1:**
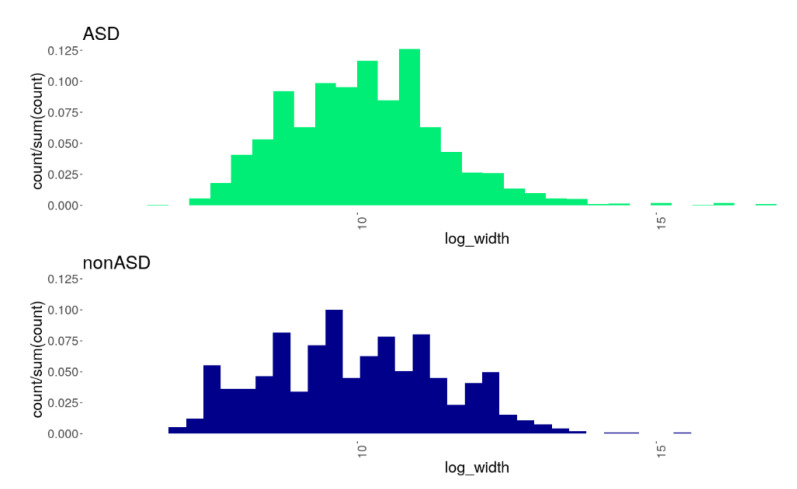
Histograms showing the distributions of CNVs of different sizes (X-axis: log-width) in the comparison groups, ASD and nonASD. A statistically significant difference in the distributions was found (the Kolmogorov–Smirnov D = 0.0934, *p* = 4.749 × 10^−6^): the ASD cohort was characterized by a wider range in CNV length with a lower prevalence of smaller CNVs and a higher prevalence of larger CNVs compared to the nonASD cohort.

Altogether 4991 CNVs across all individuals from both comparison cohorts were detected: 4084 and 907 CNV events were identified in the ASD and nonASD cohorts, respectively (Supplementary Table S3). As seen in Figure 1, the distribution of the CNV sizes differed between the comparison cohorts; the results of the Kolmogorov–Smirnov test indicated a statistical significance of this difference (D = 0.0934, *p* = 4.749 × 10^−6^). In particular, we observed a wider range in CNV length with a lower prevalence of smaller CNVs and a higher prevalence of larger CNVs in the ASD group compared to nonASD. In addition, we found a remarkable difference in the occurrence of different CNV-types between the comparison groups. In both comparison groups, deletions predominated over duplications: the deletions/duplications ratio was 2.22 and 1.48 for the ASD and nonASD cohorts, respectively. However, the predominance of deletions was more profound in the ASD group, where a significantly greater proportion of deletions was found (OR = 1.5; z = 5.365; *p* = 1.222 × 10^−7^).

### 3.3. Genome-Wide Screening of Common ASD-Associated Variants, SNPs and CNVs

**Figure 2:**
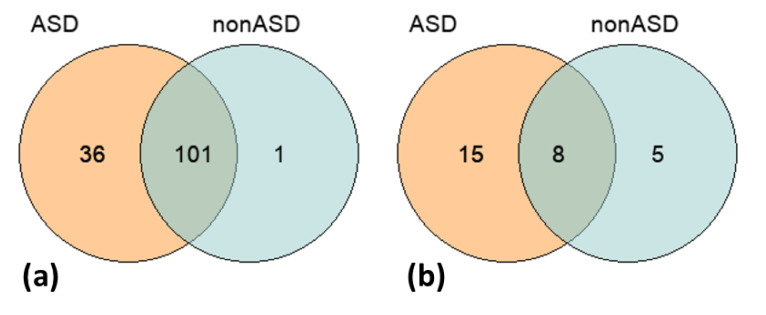
Venn diagrams represent the distributions of common ASD-associated SNPs (a) and CNVs (b) across the two comparison groups, ASD and nonASD. Both diagrams reflect a greater number of the common ASD-associated genomic variants in the ASD cohort compared to the nonASD controls.

The total unfiltered call-set of 237K SNPs identified in both comparison cohorts, ASD and nonASD, was intersected with the list of 891 common ASD-associated variants derived from the AutDB repository [34]. We found 138 of such SNPs in the studied groups (Supplementary Table S4). The distribution of these SNPs across the groups is shown in Figure 2. Although we did not find a significant overrepresentation of the common candidate variants in the ASD cohort compared to the nonASD controls, a greater number of such SNPs were identified in the discovery group—137 SNPs in ASD vs. 102 SNPs in nonASD. Additionally, Fisher’s exact test did not reveal a significant difference in the frequency of the ASD-associated SNPs between the comparison groups.

The CNV-call sets (ASD and nonASD) were also intersected with relevant data on the common ASD-related variations from the AutDB repository as per the procedures described in the Methods section. The list of overlapping CNVs was filtered according to the following criteria: localization within the same chromosome band, the same type of variation (either deletions or duplications), and the same gene content. The CNVs located on sex chromosomes and the CNVs containing HLA (human leukocyte antigen) genes, having the most extensive variability, were removed from the analysis. Detailed results of the intersection analysis are reported in Supplementary Table S5, and a summary is shown in Table 3. Altogether 29 CNVs previously associated with ASD were detected in the studied cohorts—23 and 13 CNVs in the ASD and nonASD groups, respectively, including 8 CNVs that overlapped between the comparison groups (Table 3; Figure 2). Similar to the SNP-based analysis results, despite the lack of significance in the CNV frequencies between the comparison groups, we observed a greater number of the common ASD-associated CNV-events in the ASD cohort than in the nonASD controls. 

In addition, the CNV call-sets were compared with the CNV morbidity map [59,60], or the list of structural genomic variants that have been linked to severe pediatric diseases, including developmental delays, intellectual disability, and ASD. The development delay track derived using the UCSC Genome Browser tools consists of over 29 thousand individual entries for case subjects. An intersection of these records with the ASD and non-ASD CNV call-sets resulted in 52 overlapping entries (Supplementary Table S6). It was remarkable that only four of 51 nonASD individuals (7.8%) had those CNVs; in contrast, among ASD participants, 21 of 148 individuals (14.2%) harbored CNVs linked to a developmental disorder, including the deletion CNVR6294.56 on chromosome 14, known as a very common variant [61].

### 3.4. Gene-Based and Gene Ontology-Based Analyses

Concerning the gene content of the loci harboring CNVs, we identified 1562 and 990 genes having CNVs in the ASD and nonASD cohorts, respectively (Supplementary Table S7). We performed several overrepresentation analyses (ORA) to identify particular gene ontology (GO) and human phenotype ontology (HPO) terms enriched in these gene sets. It is necessary to note that both the ASD and nonASD sets of genes harboring CNVs were extremely enriched in those encoding olfactory receptors (OR), which were assigned to the GO: olfactory receptor activity. Specifically, 173 of 1562 genes in the ASD gene-set (Fold Enrichment = 5.45; FDR = 5.61 × 10^−59^) and 167 of 990 genes in the nonASD gene-set (Fold Enrichment = 8.35; FDR = 1.88 × 10^−84^) were OR genes. Consequently, this superfamily of highly polymorphic OR genes was removed from the gene-sets prior to the ORAs.

**Figure 3:**
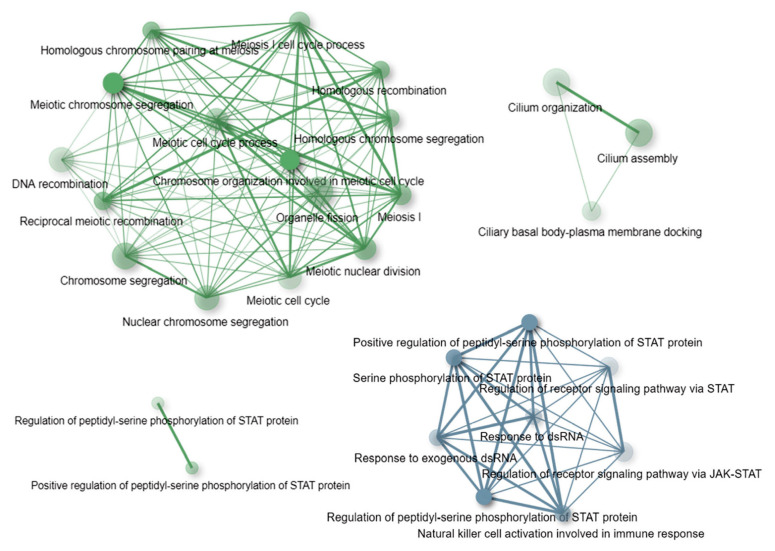
The plot shows functional categories (GO: biological process terms) significantly (at an Enrichment FDR < 10^−5^) enriched in the sets of genes harboring CNVs in the ASD cohort (green) and nonASD cohort (blue). A network indicates GO terms sharing 30% or more genes; thicker edges represent more overlapped genes. Bigger nodes correspond to larger gene sets, and darker nodes correspond to more significant enrichment FDR-values. The enrichment tests and the network constructions were performed using ShinyGO tools [62].

The ORA results are reported in the Supplementary Table S8. A graphical summary showing a top list of the most overrepresented (at an Enrichment FDR < 10^−5^) GO terms is shown in Figure 3. As can be seen in Figure 3, at the established significance threshold, the only functional category overrepresented among the genes having CNVs in the nonASD group is the JAK-STAT pathway. This signaling pathway mediates cellular transcriptional responses to cytokines and, as a consequence, is related, first of all, to the immune response. Notably, STAT protein-related pathways were also found among the GO terms overrepresented in the set of genes having CNVs in the ASD cohort (Figure 3). In turn, in comparison to the nonASD gene-set, the ASD gene-set was remarkably enriched in genes related to meiotic processes, in particular chromosome segregation, and in genes involved in the primary cilium assembly and organization (Figure 3).

The lists of genes with CNVs were also submitted to the HPO-based ORA. No significant enrichment was found for the nonASD gene-set. In contrast, the ASD gene-set was significantly enriched in a number of phenotypes related, first of all, to intellectual disability and developmental delays (Table 4). Tracking HPO-related genes in the data on the CNV distribution across individuals (Supplementary Table S9), we observed that several highlighted phenotypes had been reported in the participants’ medical records. In particular, a representative number of individuals (N = 79) harbored CNVs in the genes associated with intellectual disability. One of the ASD individuals harboring CNVs in genes related to hypertelorism manifested this phenotype according to his medical history. Two of four participants who had records on the epicanthic fold carried CNVs in the genes associated with the epicanthus. Two ASD participants were recorded as having hypotonia and one with confirmed microcephaly; however, they did not have CNVs in the genes linked to these phenotypes as per the HPO.

Important to note that enrichment of functional groups of genes does not equate to the presence of phenotypes; thus, not necessary for every patient with perturbations in particular genes to develop the corresponding phenotype. However, the top list of HPO terms in Table 4 indicates a burden of CNVs in the genes directly related to the manifestation of severe developmental issues mostly known in disorders with autosomal recessive inheritance. Remarkable, in contrast to microcephaly, we did not observe an enrichment in the HPO Macrocephaly—a clinical feature also overrepresented in individuals with ASD, which reported rates are 10–20%; see, e.g., [63,64,65]. We tend to attribute this to a power insufficiency of the enrichment analysis for this particular HPO term, for which a limited amount of associated genes (about ten) are known compared to the HPO Microcephaly, which has been reliably linked to hundreds of various genes.

## 4. Discussion

In summarizing the results of this study exploring exonic variations in a Russian cohort of children with ASD, several findings and observations need to be pointed out and discussed. First, based on the data aggregated in relevant repositories, such as AutDB [34], SFARI [37], and the CNV morbidity map of developmental delay [59,60], we tracked the genomic variants known to be implicated in ASD and related developmental disorders in the studied ASD cohort compared to the nonASD individuals from the general population of the same origin. Although below the threshold of statistical significance, an elevated prevalence of common ASD-associated genomic alterations, both SNPs and CNVs, was observed in the ASD compared to the nonASD cohorts.

Second, in comparing the CNV metrics (length, prevalence, and distribution) between the comparison groups, we observed that the ASD cohort is characterized by a higher CNV burden. Specifically, we noted a higher prevalence of larger CNV events and a remarkable predominance of deletions over duplications (about 1.5 times) in the ASD compared to the nonASD group. These observations are consistent with the consensus in the literature that CNVs are one of the most prominent sources of genetic risks for ASD. Specifically, it has been reported that CNVs, as a genomic event, are highly prevalent (i.e., observed in up to 20%) in individuals with an ASD [66]. Additionally, it has been shown that de novo CNV events occur almost five times more frequently in individuals with ASD than in unaffected siblings and controls (5–10% vs. 1–2%, respectively), and large CNVs were consistently observed in the cases with developmental delays or intellectual disability [49,51]. A recent study exploring the effect sizes of the CNV types on the development of multiple cognitive domains and overall ASD risk suggested a differential effect of deletions and duplications on different phenotypic features of ASD. Specifically, whereas both CNV types may equally affect motor skills, IQ-related cognitive deficits in ASD have been predominantly attributed to haploinsufficiency due to deletions [66]. Remarkably, our phenotype-focused enrichment tests revealed a significant overrepresentation of the comorbid phenotypes related to intellectual disabilities and developmental delays among those associated with genes having CNVs, predominantly deletions, in the studied ASD cohort. It is important to note that, in addition to these two major phenotypes, several other comorbid conditions and health problems were highlighted by the CNV gene-set enrichment analysis; for example, conditions of hypertelorism and epicanthic fold were tracked in participants’ medical records. Altogether, these observations provide additional evidence supporting the essential role of structural genomic variations in the etiology of diverse ASD phenotypes often accompanied by multiple comorbid developmental conditions.

Third, we explored potential functional outcomes of the CNV burden across the comparison groups based on tests of gene-set enrichment in the specific biological pathways that these genes control. In contrast to the nonASD controls, the list of genes harboring CNVs in the genomes of individuals with ASD showed a significant overrepresentation of gene ontologies related to meiosis and chromosome segregation, and to the biological pathways involved in primary cilium assembly and organization. Both of these biological pathways might be potentially linked to the ASD phenotype. Specifically, the former may indicate an aggravated risk of chromosomal rearrangements in ASD genomes; such rearrangements, both de novo and inherited, are known to be involved in the etiology of ASD [67,68,69]. In fact, defects and deficits in primary cilia, known to impact brain development and maturation [70,71,72,73], have been demonstrated, directly and indirectly, to contribute to ASD [74] and ASD-related phenotypes, such as Asperger syndrome [75] and Fragile X syndrome [76].

Fourth, our case-control association analysis allowed us to identify seven SNPs with a predicted deleterious effect, which showed a significantly higher prevalence in the ASD cohort compared to controls. Six genes harboring these variants: *IGLJ2*, *FAM21A*, *OR11H12*, *HIP1*, *PRAMEF10*, and *ZNF717*, have not been previously linked to ASD or ASD-related phenotypes. However, several studies have shown that *ZNF717*, *HIP1*, and several genes from the PRAME (Preferentially Expressed Antigen In Melanoma) family might be involved in cognitive development, learning disorders, and developmental disorders with autistic features [77,78]. Specifically, mutations in *PRAMEF5* and *PRAMEF7* were described in patients with delayed speech and language development, hearing deficits, and reading disability [77]. In the same study [77], a mutation in the *ZNF717* gene has been identified among 16 other rare homozygous variants in at least two families, those of patients with Joubert syndrome—a disorder characterized by autistic behavior and intellectual disability. Two reciprocal microduplications inclusive of *HIP1* have been described in three children from two unrelated families who had neurobehavioral problems: one child had an expressive language disorder, and two children had attention deficit hyperactivity disorder and manifested aggressive behavior [78]. The detection of variants within the *OR11H12* gene encoding an olfactory receptor is also not surprising, as rare and common variants in at least several OR genes have been reported in association with autism, such as *OR2M4* [79], *OR2T10* [80,81], and *OR52M1* [82].

In conclusion, despite the main limitation—a relatively small sample size—the study’s findings and observations are consistent with the growing body of evidence supporting the genetic bases of such heterogeneous disorders as ASD. We also report on several suggestive candidate genes that might be associated with ASD. Undoubtedly, follow-up research involving additional extended cohorts from the population is required to confirm the involvement of these genes in ASD; we consider these findings preliminary. To our knowledge, our study is one of the first attempts to investigate genome-wide polymorphic variants, SNPs and CNVs, in a previously understudied cohort of ASD from the Russian Federation, using a whole-exome sequencing technique.

## Figures and Tables

**Table 1:** Summarized data on clinical features of ASD individuals derived from the participants’ medical records.

Phenotype	Occurrence	Frequency (%)
Syndromes/conditions:		
Fragile X syndrome	5	2.59
Epilepsy	2	1.04
Angelman syndrome	1	0.52
Asperger’s syndrome	1	0.52
Ehlers–Danlos syndrome	1	0.52
Phelan–McDermid syndrome	1	0.52
Autoaggression	1	0.52
Macrocephaly	1	0.52
Large head (probably macrocephaly)	1	0.52
Microcephaly	1	0.52
Brachycephaly	1	0.52
Dyspepsia	1	0.52
Macrosomia	1	0.52
Hygroma	1	0.52
Neutropenia	1	0.52
Other affected anatomical systems and structures:		
Skin (hypopigmentation; «coffee» stains; intra-areolar polythelia; inverted nipples; hypertrichosis; unusual hair growth; skin prone to scarring; transverse palmar fold; hemangioma on the arm, vascular mesh on the chest)	11	5.70
Palpebral fissures (epicanthus; lower epicanthus; slightly elongated palpebral fissures; antimongoloid slanting palpebral fissures; very long eyelashes)	9	4.66
Ears (macrotia; protruding auricles; dysplastic and low-set auricles; double helix; notches on both earlobes, asymmetric auricles; deformation of the right auricle upper edge; preauricular fossa of the left ear)	8	4.15
Central Nervous System (focal cortical dysplasia; corpus callosum dysplasia; cerebral palsy, strabismus, ventricular dilatation, hippocampal hypoplasia; formations in the brain; stereotypical shaking of hands; ataxia, unusual hand movements; premature puberty)	7	3.63
Nose (short nose, slightly twisted nostrils, depressed nose bridge; upturned nose; wide nose; nasal bridge folds; low columella; wide nose bridge; sunken nose bridge)	7	3.63
Forehead (protruding frontal bones; high forehead)	6	3.11
Orbits (deep-set eyes; hypotelorism)	6	3.11
Connective tissue (joint hypermobility, skin hyperelasticity; connective tissue dysplasia; hereditary connective tissue disorder; severe myopia, marfanoid habitus)	6	3.11
Fingers/toes (clinodactyly; an additional right thumb phalanx; wide terminal phalanges of fingers and toes)	5	2.59
Face (“elfin-like” facial features; facial dysmorphisms; broad face; dysplastic face)	4	2.07
Jaws (high palate, malocclusion, uneven teeth; macrognathia; absence of two lower incisors)	4	2.07
Muscles (hypotonia, lack of tripod grasp; clumsy walking and movements; walking on tiptoes)	4	2.07
Midface (midfacial hypoplasia)	2	1.04
Torso (funnel chest, scoliosis)	2	1.04

Note. Individual data are represented in Supplementary Table S1. All syndromes have been recorded as suspected; only for two of five individuals having records on Fragile X syndrome, the syndrome has been confirmed by genetic testing.

**Table 2:** The results of the case-control, ASD vs. nonASD, association analysis of SNPs. Ten genome-wide significant ASD-associated SNPs are shown along with their pathogenicity scores and genomic annotations.

dbSNP ID	Position (hg19)	Substitution	Variant Function	Pathogenicity, C-Score ^†^	AF ^††^	P_adj_	Gene Name **	Gene Primary Function	Associated Phenotype ^⁑^
rs3121398	chr1:12954987	T > A	missense	20.30	0.1757	9.338 × 10^−3^	*PRAMEF10*	Retinoic acid receptor binding protein; RAR-mediated signaling	
rs3009023	chr3:75786628	G > C	missense	8.32	0.2378	1.260 × 10^−5^	*ZNF717*	DNA-binding transcription factor; Transcriptional regulation	
rs2918517	chr3:75786942	C > A	missense	11.55	0.2108	2.788 × 10^−4^			
rs2669761	chr10:51889683	C > A	missense	13.31	0.1882	9.828 × 10^−3^	*FAM21A*	WASH complex subunit 2A; Exocytosis	Leri–Weill dyschondrosteosis.
rs200662012	chr14:19378348	C > T	missense	20.40	0.1952	5.088 × 10^−4^	*OR11H12*	Olfactory receptor 11H12	Hereditary breast-ovarian cancer syndrome
rs200891589	chr14:19377614	G > T	missense	10.30	0.1640	1.625 × 10^−2^			
rs1167801	chr7:75176300	T > C	synonymous	10.32	0.1765	1.015 × 10^−2^	*HIP1*	Huntingtin interacting protein 1; Clathrin-mediated endocytosis and trafficking	Huntington disease; Chronic myelomonocytic leukemia; Williams–Beuren syndrome
rs1279304945	chr9:39358227	G > A	synonymous	4.35	0.1868	1.583 × 10^−3^	*SPATA31A1*	Spermatogenesis-associated protein 31A1	Familial glucocorticoid deficiency; Foramen magnum meningioma
rs1435247730	chr19:40389752	G > A	synonymous	0.14	0.1740	2.930 × 10^−2^	*FCGBP*	IgG Fc binding protein; Maintenance of the mucosal structure	Lynch syndrome; Von Willebrand disease; Congenital hypogammaglobulinemia
rs8033	chr22:23243367	T > C	synonymous	10.07	0.2460	6.870 × 10^−6^	*IGLJ2*	Immunoglobulin lambda joining protein	

Note.  ^†^ The CADD (Combined Annotation-Dependent Depletion) score [41] indicates a predicted deleterious effect of the variant on protein function: a C-Score > 20 defines a pathogenic variant, and a C-score between 10 and 20—a likely pathogenic variant [42,43]. ^††^ Allele frequencies in the ASD cohort are shown. ^⁑^ The data on the associations with phenotypes are provided based on the human diseases, MalaCards [38] and OMIM [39], and human phenotype ontologies, HPO [40], databases. ** The genes detected in this study have not been previously reported in association with ASD, as per records in the most representative relevant databases, SFARI and AutDB.

**Table 3:** The distribution of 29 common ASD-associated CNVs in the studied ASD and nonASD cohorts. Despite a lack of significant differences in the variants’ frequencies between the comparison groups, a greater number of the ASD-associated CNVs were detected in the ASD cohort compared to the nonASD controls, 23 vs. 13 CNVs.

Chromosome Band	CNV	Genes ^†^	Reference	FRQ_ASD_^††^ (N = 168)	FRQ_nonASD_ (N = 51)
1p21.1	NC_000001.11:g.103564908_103612675dup	*AMY2A*, *AMY2B*	[44]	0.0060	0
1q11–q11.2	NC_000001.11:g.120324463_ 149528945del	*SRGAP2C*	[45]	0.0060	0
1q31.3	NC_000001.11:g.196773605_196830172del	*CFHR1*, *CFHR3*	[46]	0.0714	0
1q44	NC_000001.11:g.248547045_248631695del	***OR2T10***, *OR2T11*, *OR2T29*, *OR2T34*, *OR2T35*, *OR2T5*	[44,47,48]	0.0060	0.0196
2p22.1	NC_000002.12:g.38729555_38746213dup	*GALM*, *SRSF7*	[47]	0.0060	0
2q31.2	NC_000002.12:g.178432096_178451050dup	*PRKRA*	[47]	0.0774	0
2q35	NC_000002.12:g.218818920_218956937dup	*CDK5R2*, *FEV*, *WNT10A*, *WNT6*	[49]	0.0060	0.0392
2q37.1	NC_000002.12:g.232371368_232459781dup	*ALPG*, *ALPI*, *ALPP*	[49]	0	0.0196
2q37.3	NC_000002.12:g.240678256_240774012dup	*AQP12A*, *AQP12B*, *KIF1A*	[49]	0.0179	0
3q12.2	NC_000003.12:g.100646568_100713869dup	*ADGRG7*	[47]	0.0298	0.0392
4q13.2–q13.3	NC_000004.12:g.69137075_69381445del	*UGT2B11*, *UGT2B28*	[49]	0	0.0196
6p22.2	NC_000006.12:g.26132436_26251373del	17 genes of the HIST1H gene family	[49]	0	0.0196
9q34.3	NC_000009.12:g.136887096_137799700dup	45 genes including ***GRIN1***, ***PNPLA7***, *ABCA2*, *NSMF*, and others	[47,50]	0	0.0196
11q11	NC_000011.10:g.55573260_55685410del	*OR4C11*, *OR4C15*, *OR4C16*, *OR4P4*, *OR4S2*	[48]	0.0536	0.0588
13q12.11	NC_000013.11:g.21155096_21172702dup	*SKA3*	[47]	0.0833	0.1176
13q34	NC_000013.11:g.113809317_113841915dup	*GAS6*, *TMEM255B*	[51]	0	0.0196
14q11.2	NC_000014.9:g.22773609_22780051del	***SLC7A7***	[47]	0.0060	0
14q11.2	NC_000014.9:g.19729152_19954640dup	*OR4K1*, *OR4K2*, *OR4K3*, *OR4K5*, *OR4M1*, *OR4N2*, *OR4Q3*	[52]	0.0595	0.1176
14q24.3	NC_000014.9:g.73528468_73582354del	*ACOT1*, *ACOT2*, *HEATR4*	[46]	0.0060	0.0980
14q32.33	NC_000014.9:g.106112755_106318409del	*LINC00226*	[53]	0.0060	0
14q32.33	NC_000014.9:g.105142694_105157763dup	*JAG2*	[47]	0	0.0196
17p13.1	NC_000017.11:g.10443374_10453538del	***MYH4***	[47,54]	0.0119	0
17p13.3	NC_000017.11:g.2452259_2691244dup	*METTL16*, *PAFAH1B1*	[48,53]	0.0119	0
17q21.2	NC_000017.11:g.40399039_40417791dup	*TOP2A*	[47]	0.0060	0
17q21.31	NC_000017.11:g.45616241_46136454del	*ARHGAP27*, *ARL17A*, *ARL17B*, *CRHR1*, ***KANSL1***, *CRHR1*, *MAPT*, *PLEKHM1*, *SPPL2C*, *STH*	[53,55,56,57,58]	0.0298	0
19p13.11	NC_000019.10:g.17332929_17341703dup	*ANO8*, *GTPBP3*	[47]	0.0060	0
19q13.31–q13.2	NC_000019.10:g.42738643_43237158del	*PSG1*, *PSG11*, *PSG2*, *PSG4*, *PSG5*, *PSG6*, *PSG7*, *PSG8*, *PSG9*	[53]	0.0119	0
20p12.1	NC_000020.11:g.13599877_13834151dup	*ESF1*, *NDUFAF5*, *TASP1*	[53]	0.0060	0
22q13.1	NC_000022.11:g.38963107_38989480del	*APOBEC3A*, *APOBEC3B*	[53]	0.0060	0

Note. The complete list of detected CNVs is represented in Supplementary Table S3. ^†^ The genes reported in the SFARI database as those related to ASD are marked in bold. ^††^ The CNV frequencies in the ASD (FRQ_ASD_) and nonASD (FRQ_nonASD_) groups are shown.

**Table 4:** The human phenotype ontology (HPO) terms that were significantly overrepresented among those related to genes harboring CNVs in the ASD cohort.

Human Phenotype Ontology (HPO)	Gene-Set, n	Total Genes, n	Enrichment FDR
HP:0000007 Autosomal recessive inheritance	272	2187	8.81 × 10^−22^
HP:0001249 Intellectual disability	165	1110	7.17 × 10^−20^
HP:0001263 Global developmental delay	146	1084	1.35 × 10^−13^
HP:0000252 Microcephaly	104	672	3.22 × 10^−13^
HP:0004322 Short stature	120	833	3.43 × 10^−13^
HP:0001250 Seizures	136	1047	1.37 × 10^−11^
HP:0001347 Hyperreflexia	74	442	5.94 × 10^−11^
HP:0000639 Nystagmus	95	650	1.02 × 10^−10^
HP:0001511 Intrauterine growth retardation	59	321	2.38 × 10^−10^
HP:0001252 Muscular hypotonia	80	517	3.17 × 10^−10^
HP:0000957 Cafe-au-lait spot	20	49	2.14 × 10^−9^
HP:0000340 Sloping forehead	30	110	3.13 × 10^−9^
HP:0004209 Clinodactyly of the 5th finger	46	232	3.39 × 10^−9^
HP:0100615 Ovarian neoplasm	17	36	3.39 × 10^−9^
HP:0000028 Cryptorchidism	76	508	3.59 × 10^−9^
HP:0002007 Frontal bossing	49	259	3.59 × 10^−9^
HP:0000470 Short neck	47	242	3.59 × 10^−9^
HP:0000347 Micrognathia	71	470	9.72 × 10^−9^
HP:0000486 Strabismus	78	546	1.71 × 10^−8^
HP:0000286 Epicanthus	54	318	2.15 × 10^−8^
HP:0002650 Scoliosis	83	601	2.15 × 10^−8^
HP:0006101 Finger syndactyly	35	158	2.15 × 10^−8^
HP:0000316 Hypertelorism	69	471	5.42 × 10^−8^
HP:0002119 Ventriculomegaly	47	271	1.29 × 10^−7^
HP:0000268 Dolichocephaly	28	117	1.83 × 10^−7^
HP:0001631 Atrial septal defect	40	217	3.20 × 10^−7^
HP:0003202 Skeletal muscle atrophy	44	259	7.18 × 10^−7^
HP:0000494 Downslanted palpebral fissures	46	278	7.53 × 10^−7^
HP:0000426 Prominent nasal bridge	30	141	8.25 × 10^−7^
HP:0001257 Spasticity	51	327	8.81 × 10^−7^

## Data Availability

The data that support the findings of this study are available from the authors [E.A.P., E.A.G., P.V.D., and A.K.] upon reasonable request.

## Supplementary Materials

The following are available online at https://www.mdpi.com/article/10.3390/genes13050920/s1, Figure S1: Tranches plot (SureSelect V7), Figure S2: Tranches plot (TruSeqExome), Table S1: Participants. Demographics and phenotypic data, Table S2: Summary statistics on genotyping results, provided separately for three WES data subsets prepared with three exome capture assays, Table S3: CNVs identified across all individuals from both comparison groups, ASD and nonASD, Table S4: The distribution of common ASD-associated SNPs in the studied ASD and nonASD cohorts, as per the results of an intersection analysis with relevant data sets from the AutDB repository, Table S5: Common ASD-associated CNVs detected in the studied cohorts, as per the results of an intersection analysis with relevant data sets from the AutDB repository, Table S6: CNV dataset and Developmental delay morbidity map intersection results, Table S7: List of genes located within the CNVs’ loci detected in the studied cohorts, ASD and nonASD, Table S8: Top-30 list of GO terms ranked by Enrichment P-value, which were significantly overrepresented in the sets of genes harboring CNVs in ASD and nonASD, respectively, Table S9: HPO tracking in individual data.

## References

[1] Yeargin-Allsopp M., Rice C., Karapurkar T., Doernberg N., Boyle C., Murphy C. (2003). Prevalence of autism in a US metropolitan area. JAMA.

[2] Newschaffer C.J., Croen L.A., Daniels J., Giarelli E., Grether J.K., Levy S.E., Mandell D.S., Miller L.A., Pinto-Martin J., Reaven J. (2007). The epidemiology of autism spectrum disorders. Annu. Rev. Public Health.

[3] Mpaka D.M., Okitundu D.L., Ndjukendi A.O., N’Situ A.M., Kinsala S.Y., Mukau J.E., Ngoma V.M., Kashala-Abotnes E., Ma-Miezi-Mampunza S., Vogels A. (2016). Prevalence and comorbidities of autism among children referred to the outpatient clinics for neurodevelopmental disorders. Pan. Afr. Med. J..

[4] Doshi-Velez F., Ge Y., Kohane I. (2014). Comorbidity clusters in autism spectrum disorders: An electronic health record time-series analysis. Pediatrics.

[5] Mannion A., Leader G. (2013). Comorbidity in autism spectrum disorder: A literature review. Res. Autism Spectr. Disord..

[6] Baxter A.J., Brugha T.S., Erskine H.E., Scheurer R.W., Vos T., Scott J.G. (2015). The epidemiology and global burden of autism spectrum disorders. Psychol. Med..

[7] Maenner M.J., Shaw K.A., Baio J., Washington A., Patrick M., DiRienzo M., Christensen D.L., Wiggins L.D., Pettygrove S., Andrews J.G. (2020). Prevalence of autism spectrum disorder among children aged 8 years—Autism and developmental disabilities monitoring network, 11 sites, United States, 2016. Morb. Mortal. Wkly. Rep. Surveill. Summ..

[8] Lyall K., Croen L., Daniels J., Fallin M.D., Ladd-Acosta C., Lee B.K., Park B.Y., Snyder N.W., Schendel D., Volk H. (2017). The changing epidemiology of autism spectrum disorders. Annu. Rev. Public Health.

[9] Hallmayer J., Cleveland S., Torres A., Phillips J., Cohen B., Torigoe T., Miller J., Fedele A., Collins J., Smith K. (2011). Genetic heritability and shared environmental factors among twin pairs with autism. Arch. Gen. Psychiatry.

[10] Bai D., Yip B.H.K., Windham G.C., Sourander A., Francis R., Yoffe R., Glasson E., Mahjani B., Suominen A., Leonard H. (2019). Association of genetic and environmental factors with autism in a 5-country cohort. JAMA Psychiatry.

[11] Colvert E., Tick B., McEwen F., Stewart C., Curran S.R., Woodhouse E., Gillan N., Hallett V., Lietz S., Garnett T. (2015). Heritability of autism spectrum disorder in a UK population-based twin sample. JAMA Psychiatry.

[12] Sandin S., Lichtenstein P., Kuja-Halkola R., Larsson H., Hultman C.M., Reichenberg A. (2014). The familial risk of autism. Jama.

[13] Tick B., Bolton P., Happé F., Rutter M., Rijsdijk F. (2016). Heritability of autism spectrum disorders: A meta-analysis of twin studies. J. Child Psychol. Psychiatry.

[14] Grove J., Ripke S., Als T.D., Mattheisen M., Walters R.K., Won H., Pallesen J., Agerbo E., Andreassen O.A., Anney R. (2019). Identification of common genetic risk variants for autism spectrum disorder. Nat. Genet..

[15] Havdahl A., Niarchou M., Starnawska A., Uddin M., van der Merwe C., Warrier V. (2021). Genetic contributions to autism spectrum disorder. Psychol. Med..

[16] Bourgeron T. (2016). Current knowledge on the genetics of autism and propositions for future research. Comptes Rendus Biol..

[17] Christensen D.L., Baio J., Van Naarden Braun K., Bilder D., Charles J., Constantino J.N., Daniels J., Durkin M.S., Fitzgerald R.T., Kurzius-Spencer M. (2016). Prevalence and characteristics of autism spectrum disorder among children aged 8 years—Autism and developmental disabilities monitoring network, 11 Sites, United States, 2012. Morb. Mortal. Wkly. Rep. Surveill. Summ..

[18] Tromans S., Chester V., Gemegah E., Roberts K., Morgan Z., Yao G.L., Brugha T. (2021). Autism identification across ethnic groups: A narrative review. Adv. Autism.

[19] Schott W., Tao S., Shea L. (2022). Co-occurring conditions and racial-ethnic disparities: Medicaid enrolled adults on the autism spectrum. Autism Res..

[20] Becerra T.A., von Ehrenstein O.S., Heck J.E., Olsen J., Arah O.A., Jeste S.S., Rodriguez M., Ritz B. (2014). Autism spectrum disorders and race, ethnicity, and nativity: A population-based study. Pediatrics.

[21] Morinaga M., Rai D., Hollander A.-C., Petros N., Dalman C., Magnusson C. (2020). Migration or ethnic minority status and risk of autism spectrum disorders and intellectual disability: Systematic review. Eur. J. Public Health.

[22] Da Costa G.E., Fernandes G.L., Rodrigues J.C.G., da VB Leal D.F., Pastana L.F., Pereira E.E.B., Assumpção P.P., Burbano R.M.R., dos Santos S.E.B., Guerreiro J.F. (2022). Exome evaluation of autism-associated genes in amazon american populations. Genes.

[23] Pizzo L., Jensen M., Polyak A., Rosenfeld J.A., Mannik K., Krishnan A., McCready E., Pichon O., Le Caignec C., Van Dijck A. (2019). Rare variants in the genetic background modulate cognitive and developmental phenotypes in individuals carrying disease-associated variants. Genet. Med..

[24] Andrews S. (2010). FastQC: A Quality Control Tool for High Throughput Sequence Data. http://www.bioinformatics.babraham.ac.uk/projects/fastqc/.

[25] Li H., Durbin R. (2009). Fast and accurate short read alignment with Burrows–Wheeler transform. Bioinformatics.

[26] Van der Auwera G.A., Carneiro M.O., Hartl C., Poplin R., Del Angel G., Levy-Moonshine A., Jordan T., Shakir K., Roazen D., Thibault J. (2013). From FastQ data to high confidence variant calls: The genome analysis toolkit best practices pipeline. Curr. Protoc. Bioinform..

[27] Li H. (2011). A statistical framework for SNP calling, mutation discovery, association mapping and population genetical parameter estimation from sequencing data. Bioinformatics.

[28] Wang K., Li M., Hakonarson H. (2010). ANNOVAR: Functional annotation of genetic variants from high-throughput sequencing data. Nucleic Acids Res..

[29] Klambauer G., Schwarzbauer K., Mayr A., Clevert D.-A., Mitterecker A., Bodenhofer U., Hochreiter S. (2012). MOPS: Mixture of Poissons for discovering copy number variations in next-generation sequencing data with a low false discovery rate. Nucleic Acids Research.

[30] Seshan V.E., Olshen A., DNAcopy: DNA Copy Number Data Analysis (2021). R Package Version 1.66.0. https://bioconductor.org/packages/release/bioc/html/DNAcopy.html.

[31] Purcell S., Neale B., Todd-Brown K., Thomas L., Ferreira M.A.R., Bender D., Maller J., Sklar P., de Bakker P.I.W., Daly M.J. (2007). PLINK: A tool set for whole-genome association and population-based linkage analyses. Am. J. Hum. Genet..

[32] Kumar P., Henikoff S., Ng P.C. (2009). Predicting the effects of coding non-synonymous variants on protein function using the SIFT algorithm. Nat. Protoc..

[33] Adzhubei I., Jordan D.M., Sunyaev S.R. (2014). Predicting functional effect of human missense mutations using PolyPhen-2. Curr. Protoc. Hum. Genet..

[34] Basu S.N., Kollu R., Banerjee-Basu S. (2009). AutDB: A gene reference resource for autism research. Nucleic Acids Res..

[35] Auton A., Brooks L.D., Durbin R.M., Garrison E.P., Kang H.M., Korbel J.O., Marchini J.L., McCarthy S., McVean G.A., 1000 Genomes Project Consortium (2015). A global reference for human genetic variation. Nature.

[36] Wang J., Raskin L., Samuels D.C., Shyr Y., Guo Y. (2014). Genome measures used for quality control are dependent on gene function and ancestry. Bioinformatics.

[37] Banerjee-Basu S., Packer A. (2010). SFARI Gene: An evolving database for the autism research community. Dis. Models Mech..

[38] Rappaport N., Nativ N., Stelzer G., Twik M., Guan-Golan Y., Stein T.I., Bahir I., Belinky F., Morrey C.P., Safran M. (2013). MalaCards: An integrated compendium for diseases and their annotation. Database.

[39] Hamosh A., Scott A.F., Amberger J.S., Bocchini C.A., McKusick V.A. (2005). Online Mendelian Inheritance in Man (OMIM), a knowledgebase of human genes and genetic disorders. Nucleic Acids Res..

[40] Robinson P.N., Köhler S., Bauer S., Seelow D., Horn D., Mundlos S. (2008). The human phenotype ontology: A tool for annotating and analyzing human hereditary disease. Am. J. Hum. Genet..

[41] Rentzsch P., Witten D., Cooper G.M., Shendure J., Kircher M. (2018). CADD: Predicting the deleteriousness of variants throughout the human genome. Nucleic Acids Res..

[42] Niroula A., Vihinen M. (2019). How good are pathogenicity predictors in detecting benign variants?. PLoS Comput. Biol..

[43] Kircher M., Witten D.M., Jain P., O’Roak B.J., Cooper G.M., Shendure J. (2014). A general framework for estimating the relative pathogenicity of human genetic variants. Nat. Genet..

[44] Lintas C., Picinelli C., Piras I.S., Sacco R., Brogna C., Persico A.M. (2017). Copy number variation in 19 Italian multiplex families with autism spectrum disorder: Importance of synaptic and neurite elongation genes. Am. J. Med. Genet. B Neuropsychiatr. Genet..

[45] Wang S., Mandell J.D., Kumar Y., Sun N., Morris M.T., Arbelaez J., Nasello C., Dong S., Duhn C., Zhao X. (2018). De novo sequence and copy number variants are strongly associated with tourette disorder and implicate cell polarity in pathogenesis. Cell Rep..

[46] Celestino-Soper P.B., Shaw C.A., Sanders S.J., Li J., Murtha M.T., Ercan-Sencicek A.G., Davis L., Thomson S., Gambin T., Chinault A.C. (2011). Use of array CGH to detect exonic copy number variants throughout the genome in autism families detects a novel deletion in TMLHE. Hum. Mol. Genet..

[47] Krumm N., Turner T.N., Baker C., Vives L., Mohajeri K., Witherspoon K., Raja A., Coe B.P., Stessman H.A., He Z.X. (2015). Excess of rare, inherited truncating mutations in autism. Nat. Genet..

[48] Girirajan S., Brkanac Z., Coe B.P., Baker C., Vives L., Vu T.H., Shafer N., Bernier R., Ferrero G.B., Silengo M. (2011). Relative burden of large CNVs on a range of neurodevelopmental phenotypes. PLoS Genet..

[49] Pinto D., Delaby E., Merico D., Barbosa M., Merikangas A., Klei L., Thiruvahindrapuram B., Xu X., Ziman R., Wang Z. (2014). Convergence of genes and cellular pathways dysregulated in autism spectrum disorders. Am. J. Hum. Genet..

[50] Yatsenko S.A., Hixson P., Roney E.K., Scott D.A., Schaaf C.P., Ng Y.T., Palmer R., Fisher R.B., Patel A., Cheung S.W. (2012). Human subtelomeric copy number gains suggest a DNA replication mechanism for formation: Beyond breakage-fusion-bridge for telomere stabilization. Hum. Genet..

[51] Pinto D., Pagnamenta A.T., Klei L., Anney R., Merico D., Regan R., Conroy J., Magalhaes T.R., Correia C., Abrahams B.S. (2010). Functional impact of global rare copy number variation in autism spectrum disorders. Nature.

[52] AlAyadhi L.Y., Hashmi J.A., Iqbal M., Albalawi A.M., Samman M.I., Elamin N.E., Bashir S., Basit S. (2016). High-resolution SNP genotyping platform identified recurrent and novel CNVs in autism multiplex families. Neuroscience.

[53] Kaminsky E.B., Kaul V., Paschall J., Church D.M., Bunke B., Kunig D., Moreno-De-Luca D., Moreno-De-Luca A., Mulle J.G., Warren S.T. (2011). An evidence-based approach to establish the functional and clinical significance of copy number variants in intellectual and developmental disabilities. Genet. Med..

[54] O’Roak B.J., Vives L., Girirajan S., Karakoc E., Krumm N., Coe B.P., Levy R., Ko A., Lee C., Smith J.D. (2012). Sporadic autism exomes reveal a highly interconnected protein network of de novo mutations. Nature.

[55] Sajan S.A., Fernandez L., Nieh S.E., Rider E., Bukshpun P., Wakahiro M., Christian S.L., Rivière J.B., Sullivan C.T., Sudi J. (2013). Both rare and de novo copy number variants are prevalent in agenesis of the corpus callosum but not in cerebellar hypoplasia or polymicrogyria. PLoS Genet..

[56] Asadollahi R., Oneda B., Joset P., Azzarello-Burri S., Bartholdi D., Steindl K., Vincent M., Cobilanschi J., Sticht H., Baldinger R. (2014). The clinical significance of small copy number variants in neurodevelopmental disorders. J. Med. Genet..

[57] Di Gregorio E., Riberi E., Belligni E.F., Biamino E., Spielmann M., Ala U., Calcia A., Bagnasco I., Carli D., Gai G. (2017). Copy number variants analysis in a cohort of isolated and syndromic developmental delay/intellectual disability reveals novel genomic disorders, position effects and candidate disease genes. Clin. Genet..

[58] Munnich A., Demily C., Frugère L., Duwime C., Malan V., Barcia G., Vidal C., Throo E., Besmond C., Hubert L. (2019). Impact of on-site clinical genetics consultations on diagnostic rate in children and young adults with autism spectrum disorder. Mol. Autism.

[59] Coe B.P., Witherspoon K., Rosenfeld J.A., van Bon B.W., Vulto-van Silfhout A.T., Bosco P., Friend K.L., Baker C., Buono S., Vissers L.E. (2014). Refining analyses of copy number variation identifies specific genes associated with developmental delay. Nat. Genet..

[60] Cooper G.M., Coe B.P., Girirajan S., Rosenfeld J.A., Vu T.H., Baker C., Williams C., Stalker H., Hamid R., Hannig V. (2011). A copy number variation morbidity map of developmental delay. Nat. Genet..

[61] Conrad D.F., Pinto D., Redon R., Feuk L., Gokcumen O., Zhang Y., Aerts J., Andrews T.D., Barnes C., Campbell P. (2010). Origins and functional impact of copy number variation in the human genome. Nature.

[62] Ge S.X., Jung D., Yao R. (2020). ShinyGO: A graphical gene-set enrichment tool for animals and plants. Bioinformatics.

[63] Fidler D.J., Bailey J.N., Smalley S.L. (2000). Macrocephaly in autism and other pervasive developmental disorders. Dev. Med. Child Neurol..

[64] Fombonne E., Rogé B., Claverie J., Courty S., Frémolle J. (1999). Microcephaly and macrocephaly in autism. J. Autism Dev. Disord..

[65] Lainhart J.E., Bigler E.D., Bocian M., Coon H., Dinh E., Dawson G., Deutsch C.K., Dunn M., Estes A., Tager-Flusberg H. (2006). Head circumference and height in autism: A study by the collaborative program of excellence in autism. Am. J. Med. Genet. Part A.

[66] Douard E., Zeribi A., Schramm C., Tamer P., Loum M.A., Nowak S., Saci Z., Lord M.P., Rodríguez-Herreros B., Jean-Louis M. (2021). Effect Sizes of deletions and duplications on autism risk across the genome. Am. J. Psychiatry.

[67] Sener E.F. (2014). Association of copy number variations in autism spectrum disorders: A systematic review. Chin. J. Biol..

[68] Tabet A.-C., Verloes A., Pilorge M., Delaby E., Delorme R., Nygren G., Devillard F., Gérard M., Passemard S., Héron D. (2015). Complex nature of apparently balanced chromosomal rearrangements in patients with autism spectrum disorder. Mol. Autism.

[69] Rylaarsdam L., Guemez-Gamboa A. (2019). Genetic causes and modifiers of autism spectrum disorder. Front. Cell. Neurosci..

[70] Park S.M., Jang H.J., Lee J.H. (2019). Roles of primary cilia in the developing brain. Front. Cell. Neurosci..

[71] Guemez-Gamboa A., Coufal N.G., Gleeson J.G. (2014). Primary cilia in the developing and mature brain. Neuron.

[72] Guo J., Higginbotham H., Li J., Nichols J., Hirt J., Ghukasyan V., Anton E.S. (2015). Developmental disruptions underlying brain abnormalities in ciliopathies. Nat. Commun..

[73] Guo J., Otis J.M., Higginbotham H., Monckton C., Cheng J., Asokan A., Mykytyn K., Caspary T., Stuber G.D., Anton E.S. (2017). Primary cilia signaling shapes the development of interneuronal connectivity. Dev. Cell.

[74] Trulioff A., Ermakov A., Malashichev Y. (2017). Primary Cilia as a possible link between left-right asymmetry and neurodevelopmental diseases. Genes.

[75] Kondziella D., Lycke J. (2008). Autism spectrum disorders: Does cilia dysfunction in embryogenesis play a role?. Acta Neuropsychiatr..

[76] Lee B., Panda S., Lee H.Y. (2020). Primary ciliary deficits in the dentate gyrus of fragile X syndrome. Stem Cell Rep..

[77] Lei H., Yan Z., Sun X., Zhang Y., Wang J., Ma C., Xu Q., Wang R., Jarvis E.D., Sun Z. (2017). Axon guidance pathways served as common targets for human speech/language evolution and related disorders. Brain Lang..

[78] Ramocki M.B., Bartnik M., Szafranski P., Kołodziejska K.E., Xia Z., Bravo J., Miller G.S., Rodriguez D.L., Williams C.A., Bader P.I. (2010). Recurrent distal 7q11.23 Deletion including HIP1 and YWHAG identified in patients with intellectual disabilities, epilepsy, and neurobehavioral problems. Am. J. Hum. Genet..

[79] Kuo P.H., Chuang L.C., Su M.H., Chen C.H., Chen C.H., Wu J.Y., Yen C.J., Wu Y.Y., Liu S.K., Chou M.C. (2015). Genome-wide association study for autism spectrum disorder in Taiwanese Han population. PLoS ONE.

[80] Iossifov I., O’Roak B.J., Sanders S.J., Ronemus M., Krumm N., Levy D., Stessman H.A., Witherspoon K.T., Vives L., Patterson K.E. (2014). The contribution of de novo coding mutations to autism spectrum disorder. Nature.

[81] Codina-Solà M., Rodríguez-Santiago B., Homs A., Santoyo J., Rigau M., Aznar-Laín G., Del Campo M., Gener B., Gabau E., Botella M.P. (2015). Integrated analysis of whole-exome sequencing and transcriptome profiling in males with autism spectrum disorders. Mol. Autism.

[82] Sanders S.J., He X., Willsey A.J., Ercan-Sencicek A.G., Samocha K.E., Cicek A.E., Murtha M.T., Bal V.H., Bishop S.L., Dong S. (2015). Insights into autism spectrum disorder genomic architecture and biology from 71 risk loci. Neuron.

