# Supplementary material for: A Study of the Genomic Variations Associated with Autistic Spectrum Disorders in a Russian Cohort of Patients Using Whole-Exome Sequencing
Source: Genes (Basel). 2022 May 20;13(5):920. doi: 10.3390/genes13050920 (PMC9141003; doi:10.3390/genes13050920)
Supplement: Supplementary file 1 [file genes-13-00920-s001.zip › Figure S1 and Figure S2.pdf]

(**A**)

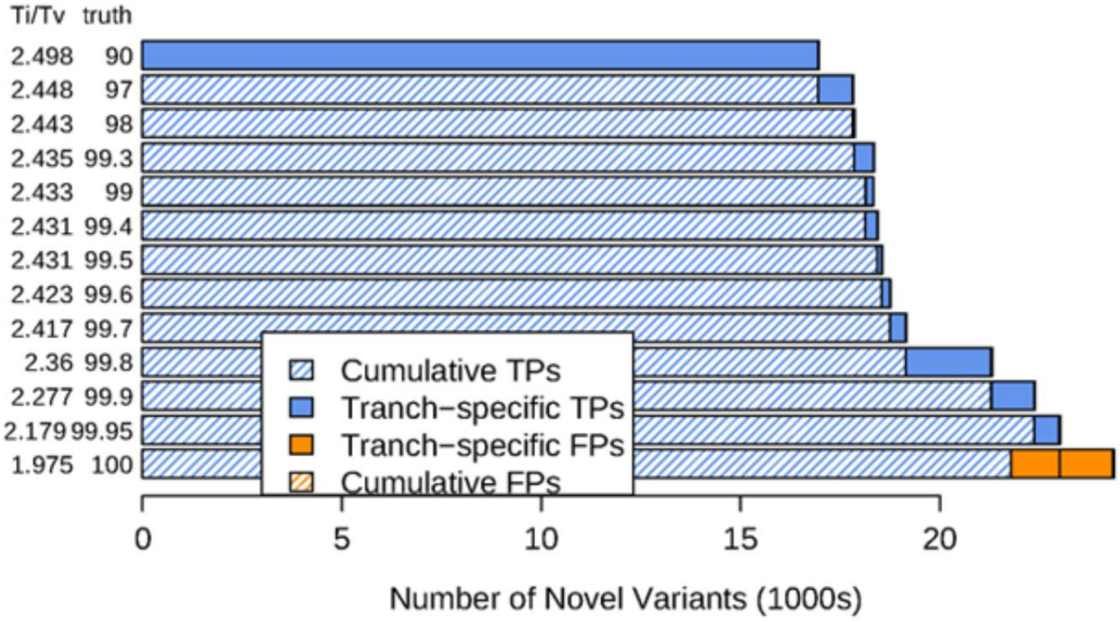

(**B**)

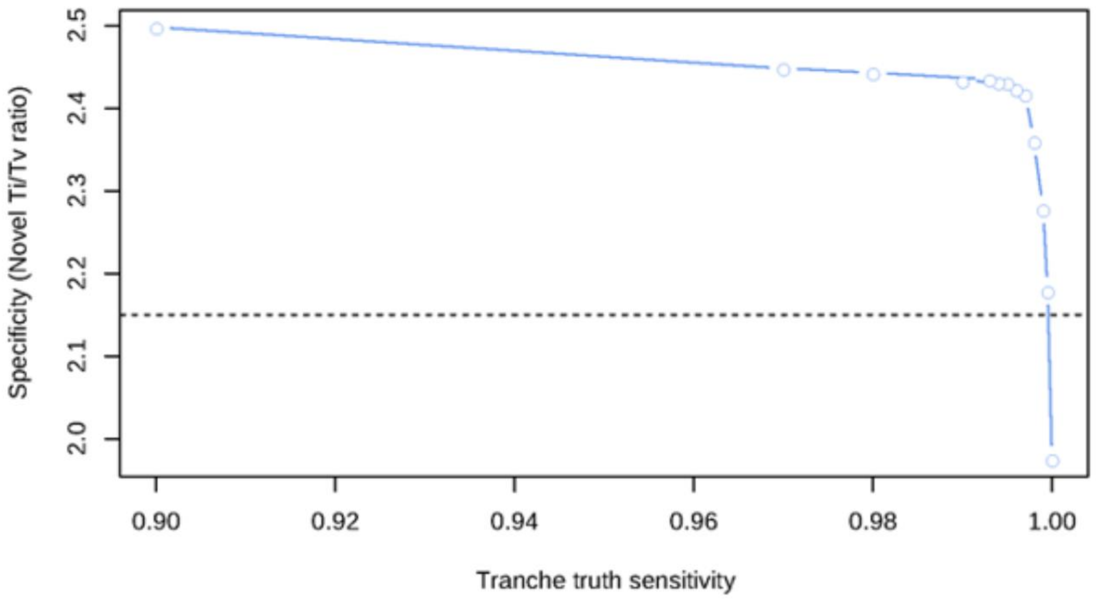

Figure S1. Tranches plot (SureSelect V7). (**A**)Tranches plot (SureSelect V7), (**B**)Sensitivity-specificity relation (SureSelect V7).

(**A**)

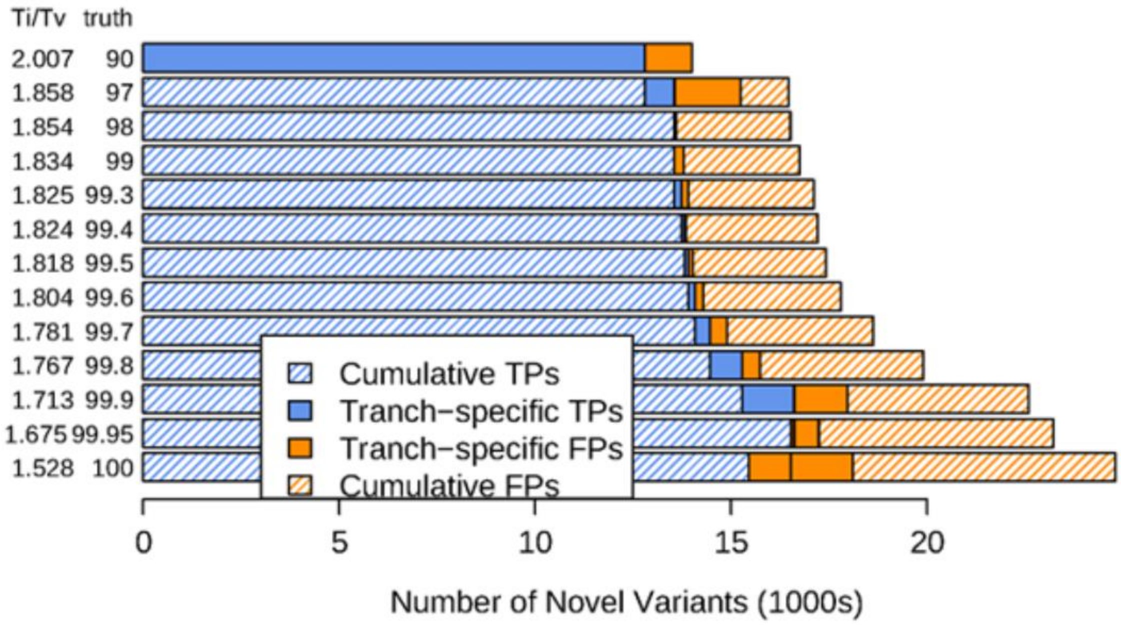

(**B**)

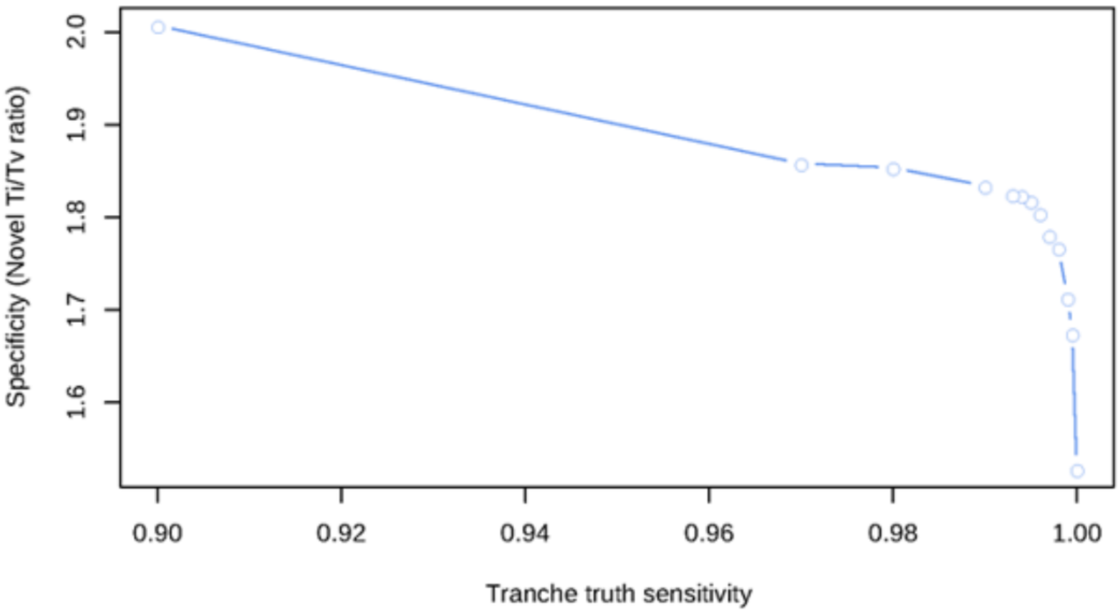

Figure S2. Tranches plot (TruSeqExome). (**A**) Tranches plot (TruSeq DNA Exome), (**B**) Sensitivity-specificity relation (TruSeq DNA Exome).
