# Supplementary material for: A Study of the Genomic Variations Associated with Autistic Spectrum Disorders in a Russian Cohort of Patients Using Whole-Exome Sequencing
Source: Genes (Basel). 2022 May 20;13(5):920. doi: 10.3390/genes13050920 (PMC9141003; doi:10.3390/genes13050920)
Supplement: Supplementary file 1 [file genes-13-00920-s001.zip › Table S2.pdf]

Table S2. Summary statistics on genotyping results, provided separately for three WES data subsets prepared with three exome capture assays.

QC metric	ASD (TruSeq)	ASD (SureSelect V7)	nonASD (SureSelect V6)	Expected values
# SNP Total	145653	128432	95964	–
# SNP per sample	~31762 (sd 1397.8)	~23104.5 (sd 429.2)	~24186.5 (sd 516.8)	~25000 (1000 Genomes, 2015)
nHet/ nNonRefHom	1.60 (sd 0.06)	1.65 (sd 0.06)	1.59 (sd 0.08)	~1.6 (Wang et al., 2014)
Ti/Tv	2.73 (sd 0.04)	2.94 (sd 0.02)	2.93 (sd 0.08)	~3.0 (Wang et al., 2014)
# CNV Total	2525	1559	907	–
